# The *Strongyloides stercoralis*-hookworms association as a path to the estimation of the global burden of strongyloidiasis: A systematic review

**DOI:** 10.1371/journal.pntd.0008184

**Published:** 2020-04-13

**Authors:** Pedro E. Fleitas, Marina Travacio, Helena Martí-Soler, M. Eugenia Socías, Walter R. Lopez, Alejandro J. Krolewiecki

**Affiliations:** 1 Universidad Nacional de Salta, Instituto de Investigaciones de Enfermedades Tropicales/CONICET, Orán, Salta, Argentina; 2 Universidad Nacional de Salta, Cátedra de Química Biológica, Facultad de Ciencias Naturales, Salta, Salta, Argentina; 3 Universidad de Buenos Aires. Facultad de Farmacia y Bioquímica, Cátedra de Química General e Inorgánica, Buenos Aires, Argentina; 4 Universitat de Barcelona, ISGlobal, Hospital Clínic, Barcelona, Cataluña, Spain; 5 University of British Columbia, British Columbia Centre on Substance Use, Department of Medicine, Vancouver, BC, Canada; Yale Child Health Research Center, UNITED STATES

## Abstract

Soil-transmitted helminths (STH) represent a significant public health problem. However, *Strongyloides stercoralis* is not yet integrated into the control strategy against STH, given limitations to accurately assess its burden. Considering that *S*. *stercoralis* shares biological and epidemiological characteristics with hookworms, we describe a new approach for an improved estimation of the burden of infections by *S*. *stercoralis* based on the prevalence and burden of hookworms and the relationship between these species. A systematic review of publications reporting prevalence rates for *S*. *stercoralis* and hookworms was carried out. The data was classified into two categories: 1) “Community”, with surveys including all age groups, and 2) “SAC”, with surveys limited to school-aged children. The relationship between S. *stercoralis* and hookworms was characterized in order to estimate the global burden of *S*. *stercoralis* infections. The study is registered in PROSPERO (CRD42019131127). Spearman correlation coefficient between *S*. *stercoralis* and hookworms was estimated and the global burden of *S*. *stercoralis* infections was estimated using a regression model. A total of 119 articles were included, and a significant positive correlation between the burden of *S*. *stercoralis* and hookworms was identified. Spearman’s coefficient for Community surveys was 0.94 and for SAC surveys it was 0.63. Based on the linear model, the global burden of *S*. *stercoralis* infections was estimated at 386 million (95%CI 324–449 million) people, including 22 million (95%CI 20–24 million) SAC. The significant relationship between *S*. *stercoralis* and hookworms allows an estimation of the global burden of *S*. *stercoralis* infections in most epidemiologic settings using hookworm burden and justifies the search of integrated control activities.

**Trial registration:**

PROSPERO (registration code CRD42019131127).

## Author summary

Evidence on epidemiologic similarities between *Strongyloides stercoralis* and hookworms was confirmed by our group. A systematic bibliographic search of surveys published between 2001 and 2018 was carried out. Results suggested that the prevalence and burden of infections by hookworms could be used as a proxy to estimate *S*. *stercoralis* infections. This approach is based on a linear relationship between the burden of these species. In addition, the models and linearity showed a better fit for surveys using diagnostic methods with high sensitivity for both species. Our estimate of 386 million people infected with *S*. *stercoralis* globally, provides an update of the burden *of S*. *stercoralis*. This implies an estimate of almost four times higher than what was estimated previously. Our approach allows evidence-based approximation to the estimations of the global burden of *S*. *stercoralis* infections, for different age groups and geographic regions. This information highlights the hidden burden of *S*. *stercoralis* while also contributing in the estimation of the potential need for ivermectin for the treatment of neglected tropical diseases.

## Introduction

Soil-transmitted helminths (STH) are intestinal parasites that need to fulfill part of their life cycle in soil. This group of parasites represents a significant public health problem in terms of prevalence and morbidity, prompting a call from the World Health Assembly in 2001 for their control [1]. STH of relevance to human health currently included in the World Health Organization (WHO) guided strategy for the control of STH as a public health problem are *Ascaris lumbricoides*, *Trichuris trichiura* and the hookworms *Necator americanus* and *Ancylostoma duodenale*. According to WHO, more than 800 million children around the world concentrated in the most impoverished communities in tropical and sub-tropical areas, should be targeted for preventive chemotherapy through mass drug administration (MDA) of albendazole or mebendazole [2].

*Strongyloides stercoralis* is an intestinal helminth parasite of importance in human disease that also includes a soil passage in its life cycle, although it has the capacity for internal reproduction and amplification within the human host [3], but is not yet targeted by the control strategy against STH; although the recently issued list of targets for STH control programs by WHO includes the establishment of an efficient strongyloidiasis control program in school age children (SAC) by 2030 [4]. There is a lack of epidemiological information on *S*. *stercoralis* with its burden of morbidity and global prevalence largely unknown [5,6]. The main cause for this lack of information is the difficulty in diagnosing *S*. *stercoralis* in comparison with the other STH. Reported prevalence estimates range from 30 to 100 million people infected in the world, however this information originates from outdated reports containing estimates based on scarce evidence [7,8]. Moreover, these figures were mostly obtained with laboratory methods inadequate for the diagnosis of *S*. *stercoralis*, long before large scale surveys were implemented.

*S*. *stercoralis* and hookworms share biological and epidemiological characteristics including an overlapping geographic distribution [9–11]. For these species, the infection occurs when larvae in soils contaminated with fecal matter penetrate the skin. In addition, hookworms and *S*. *stercoralis* infections have a similar age distribution range [6]. Global estimates of population infected with hookworm are between 212 and 480 million people [12,13]. Despite these biological similarities, pharmacologic therapies and control strategies for these species of STH differ. Albendazole and alternatively mebendazole are the drugs of choice against hookworms, and ivermectin (IVM) is the preferred therapy against *S*. *stercoralis* [14]. With the inclusion of IVM in the latest edition of the Essential Medicines List of WHO for the treatment of STH [15], the growing list of Neglected Tropical Diseases (NTDs) targeted to benefit from the distribution of IVM sets a new stage for the demand of this drug, currently available through the Mectizan Donation Program for the treatment of onchocerciasis and lymphatic filariasis, but excluding other indications [16].

Given the increasing awareness of *S*. *stercoralis* as a STH deserving consideration as part of the global strategy against STH, we aimed to investigate whether hookworm burden could serve as proxy to estimate *S*. *stercoralis* burden, and contribute to the estimation of the potential global demand of IVM for NTDs control programs.

## Methods

### Systematic literature search

This systematic review was carried out in accordance with the instructions of the Preferred Reporting Items for Systematic Reviews and Meta-Analyses (PRISMA) [17], and the Guidelines for Accurate and Transparent Health Estimates Reporting (GATHER) [18]. The protocol for this review was registered with PROSPERO (registration code CRD42019131127). The complete PRISMA and GATHER checklists can be found in the Supporting information (S1 Table and S2 Table). A comprehensive search of the literature was conducted using the following databases: Embase, Medline, CAB, LILACS, ScieLO, Cochrane Library, Cochrane Central Register of Controlled Trials, WHO International Clinical Trials Registry Platform, and PROSPERO. The search was conducted from June 6 to June 25, 2018. The search strategy included: strongyloid* OR “S stercoralis” OR “S. stercoralis” OR estrongyloid* OR estrongiloid* AND “hook worm” OR hook-worm OR hookworm OR ancylostom* OR necator* OR uncinaria*. An example of the search strategy can be found in the Supporting information (S1 File).

The scope of the systematic literature review was defined based on the following criteria:

1. Population: Human community and school-based population surveys. Inpatient and outpatient populations, whether symptomatic or not, as well as hospital and laboratory records were excluded.
2. Outcomes: Studies reporting the prevalence of both *S*. *stercoralis* and hookworm with appropriate diagnostic methods, particularly for *S*. *stercoralis* were included.
3. Study design: Baseline pre-intervention surveys were included. Reviews, case series, case studies, laboratory database analysis, diagnostic studies and surveys from areas with active MDA programs were excluded.

Articles published in English, Spanish, French, Italian and Portuguese, between 2001 and June 2018 were considered for this review.

### Study selection and data extraction

Two study investigators reviewed independently all the titles and abstracts from references identified during the literature search to be potentially relevant to the research question. The references selected after this screening were reviewed in full by two different study investigators to determine final eligibility status. For all eligible studies, data on study characteristics, patient characteristics and outcomes were extracted in duplicate by two investigators. The study characteristics that were extracted included: author, year of publication, country, study design, population, sample size, diagnostic methods used, prevalence and burden of *S*. *stercoralis*, hookworms, *Ascaris lumbricoides* (when available) and *Trichuris trichiura* (when available). These data were entered manually into an Excel spreadsheet. Any discrepancies identified in each step of study selection and data extraction were resolved through discussion and when discrepancies could not be resolved, a third independent reviewer was consulted.

### Risk of bias

To ascertain the validity of eligible studies, two reviewers working independently and with adequate reliability determined the adequacy of subject selection from every survey included in the analysis in order to obtain representative samples. To prevent the risk of underestimating the prevalence of STH, particularly for *S*. *stercoralis*, diagnostic methods were classified as high/low sensitivity methods for *S*. *stercoralis* and hookworms.

### Data analysis

Surveys were classified into two categories according to the target population: 1) “Community”, that includes all age ranges, and 2) “SAC”, representing school-aged children. For each category, two subcategories were analyzed according to the sensitivity of the diagnostic methods used: 1) High Sensitivity (High-S), diagnostic approach including methods that report a high sensitivity for *S*. *stercoralis*, and hookworms (serology for *S*. *stercoralis*, PCR, qPCR, Harada Mori, Baermann, agar plate, and centrifugation/concentration without conservatives), and 2) Low Sensitivity (Low-S), diagnostic methods that report lower sensitivity techniques for *S*. *stercoralis*, and/or hookworm (centrifugation/concentration with preservatives, spontaneous sedimentation, McMaster, Kato Katz, FLOTAC, and Mini FLOTAC).

We estimated weighted Spearman’s correlation between the burdens of *S*. *stercoralis* and hookworms and used cubic-spline to identify their relationship. Finally, the data was analyzed using weighted regression, where the weights were defined according to the sample size of each survey. In addition, Cook’s distance was used to find influential outliers. The final model was selected according to the Akaike information criteria (AIC). We performed a cross-validation method in order to avoid over-optimistic expectations of prediction model performance. Statistical analyses were done using R software (version 3.5.3) [19].

## Results

### Summary of the systematic review

**Fig 1:**
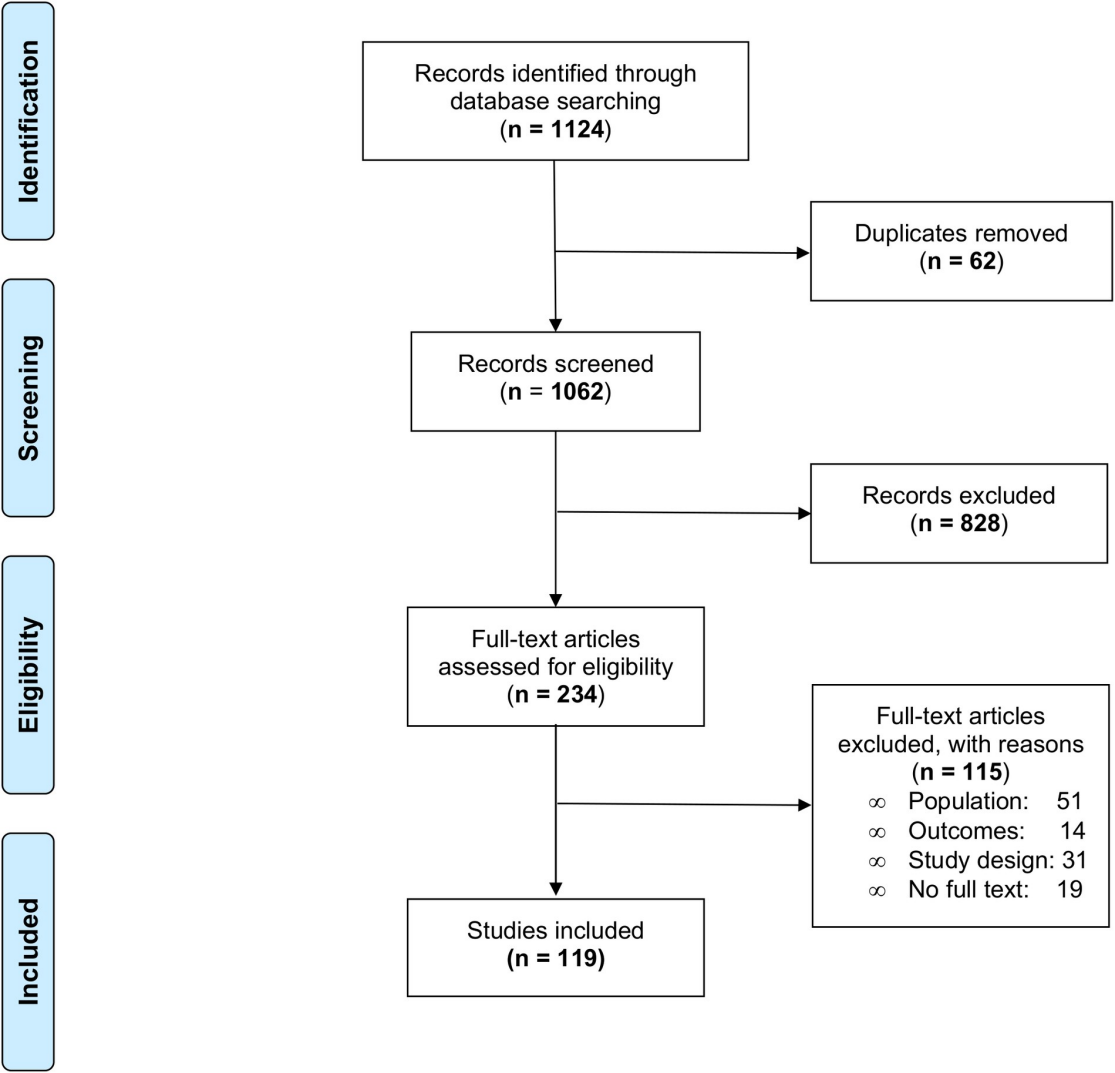
PRISMA (preferred reporting items for systematic reviews and meta-analyses) flow diagram of systematic literature search.

**Fig 2:**
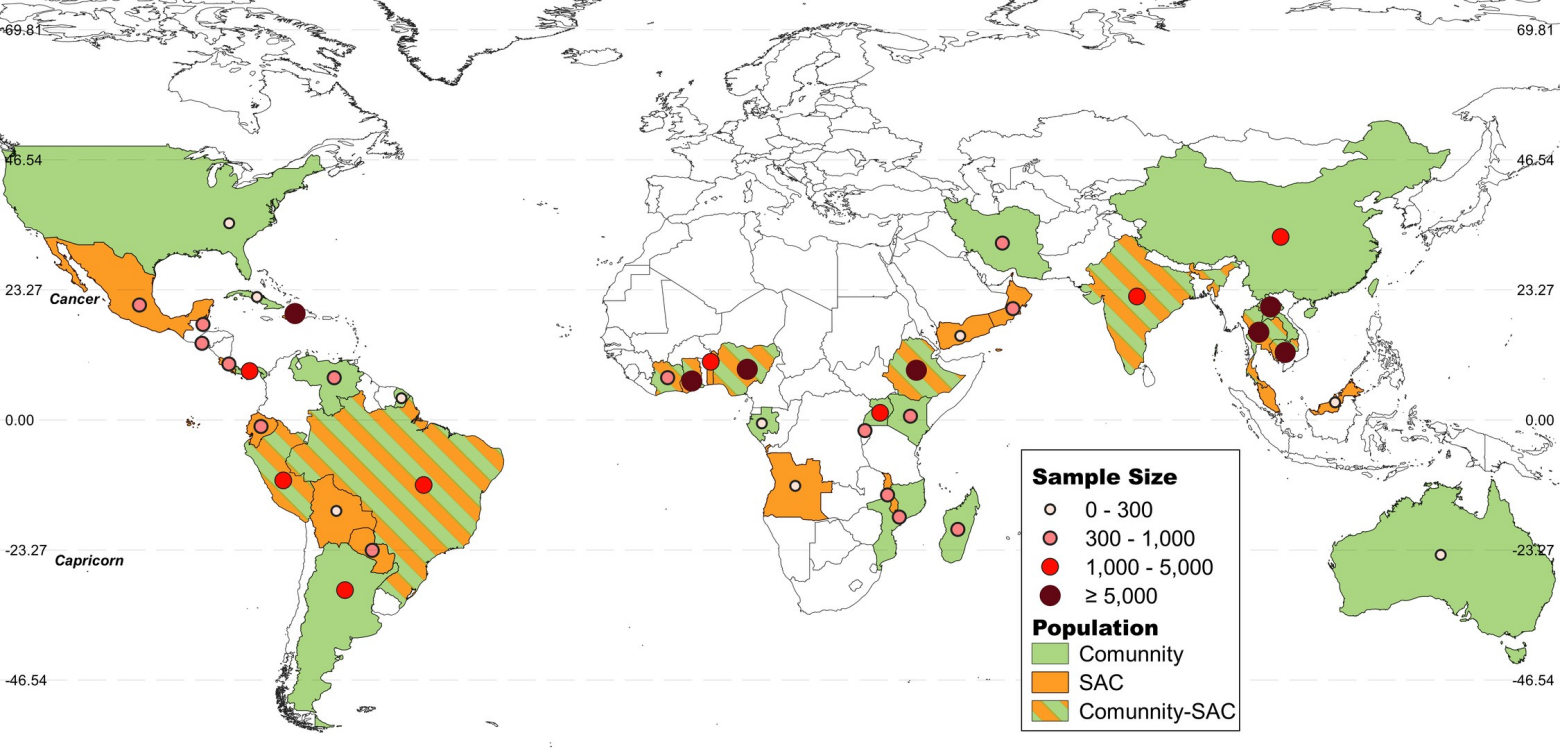
Map of the geographical distribution of the included surveys (n = 127) [20]. The size of each circle is proportional to the total number of surveyed individuals in each country. Countries are represented in green (community based surveys) or orange (school-aged children). Countries with both type of surveys are represented in green and orange bars.

The search strategy yielded 1124 publications across the nine databases. After the initial screening based on titles and abstracts, 890 records were excluded, leaving 234 full-text research articles to be evaluated for eligibility. Of those 234 papers assessed, 19 did not have a full text available, 51 did not meet the population criteria, 14 did not meet the outcomes criteria, and 31 did not meet the study design criteria. Hence, a total of 119 articles, presenting results from 127 different surveys (93958 surveyed individuals) were included (Fig 1). Forty-seven (37%) were from the Americas, 42 (33%) from Africa, 18 (14%) from South East Asia, 17 (13%) from the Western Pacific region and 3 (3%) from the Eastern Mediterranean region (Fig 2). The complete list of included articles with the extracted data can be found in the Supporting information (S3 Table and S2 File).

**Fig 3:**
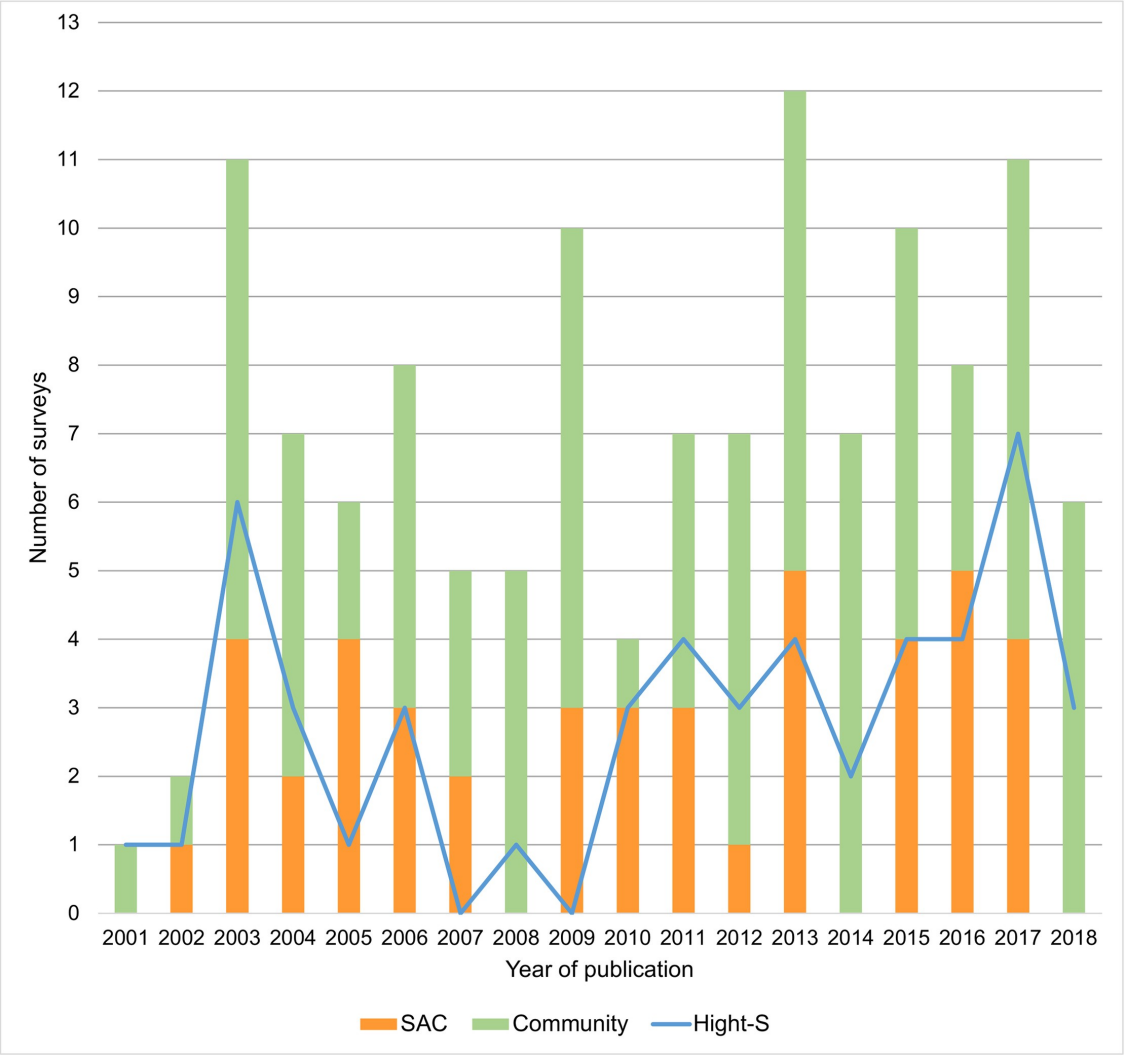
Number of surveys per year. Orange bars represent the number of surveys in school-aged children (SAC) population. Green bars represent the number of surveys of the Community population. The blue line represents the total number of surveys with highly sensitive diagnostic methods (High-S).

Out of the 127 surveys, 83 (65%) corresponded to Community based surveys including 36 (43%) High-S and 47 (57%) Low-S diagnostic methods; 44 (35%) corresponded to SAC surveys including 14 (32%) High-S methods and 30 (68%) Low-S methods. The number of surveys per year for the community and SAC categories and for the High-S subcategory are presented in Fig 3.

### Correlation among STH species

Positive correlation between the *S*. *stercoralis* and hookworms burden was identified for both populations, Community and SAC. The estimated Spearman’s correlation was 0.94 for all Community surveys and 0.63 for all SAC surveys.

On the other hand, the correlation between the burden of *S*. *stercoralis* and *A*. *lumbricoides* and between *S*. *stercoralis* and *T*. *trichiura* was 0.24 and 0.42, respectively. In addition, a positive correlation was found between *A*. *lumbricoides* and *T*. *trichiura*, with an estimated Spearman’s coefficient of 0.78.

### Regression analysis

Out of the 83 community based surveys included in the systematic review, the marked influence on the regression analysis of a survey, with 20250 surveyed individuals from Ghana (significatively higher than the average of 585 for all other surveys), was identified by Cook’s distance as an outlier (Survey #26 in S3 Table); and was therefore excluded from the regression analysis.

**Fig 4:**
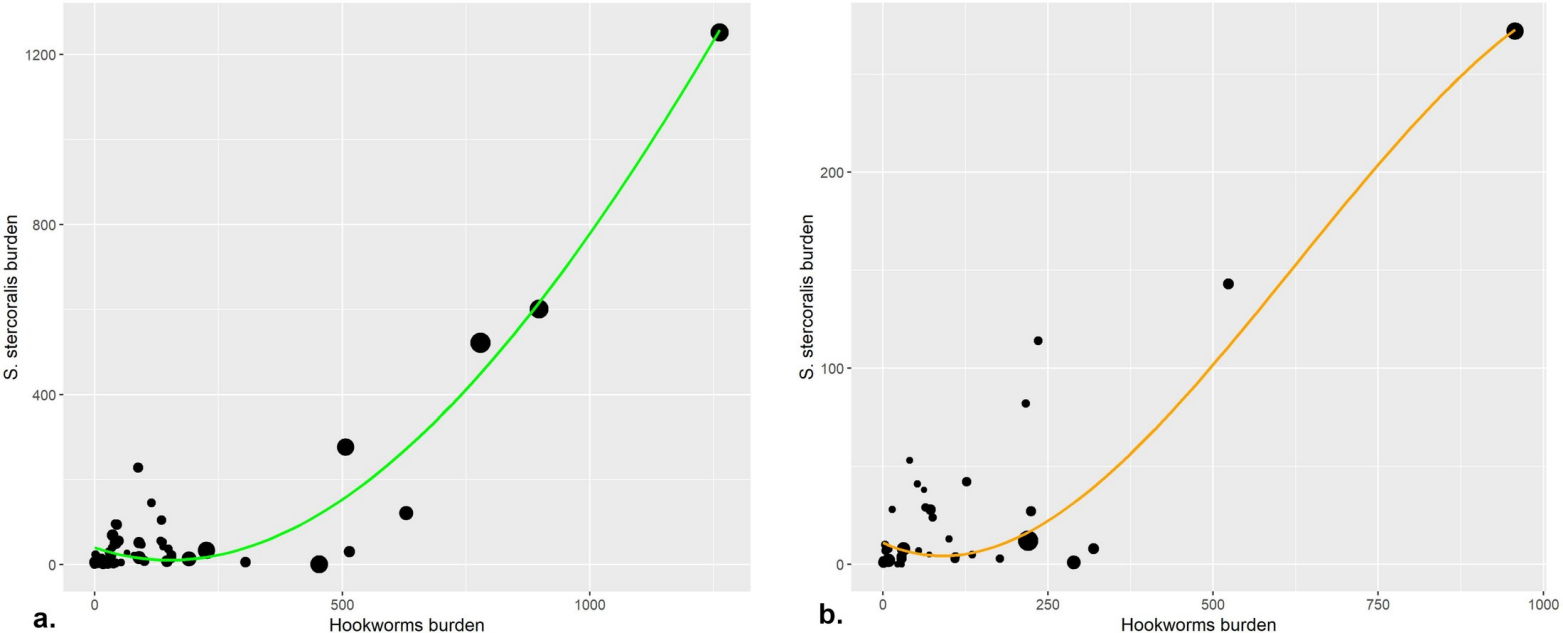
Spline of different surveys weighted by sample size. a. In black dots and green line all Community based surveys. b. In black dots and orange line: school-aged children (SAC) based surveys. The size of all dots is proportional to the sample size.

In the exploratory analysis, the shape of the relationship between the burden of *S*. *stercoralis* and hookworm was described for both types of surveys (Fig 4).

**Fig 5:**
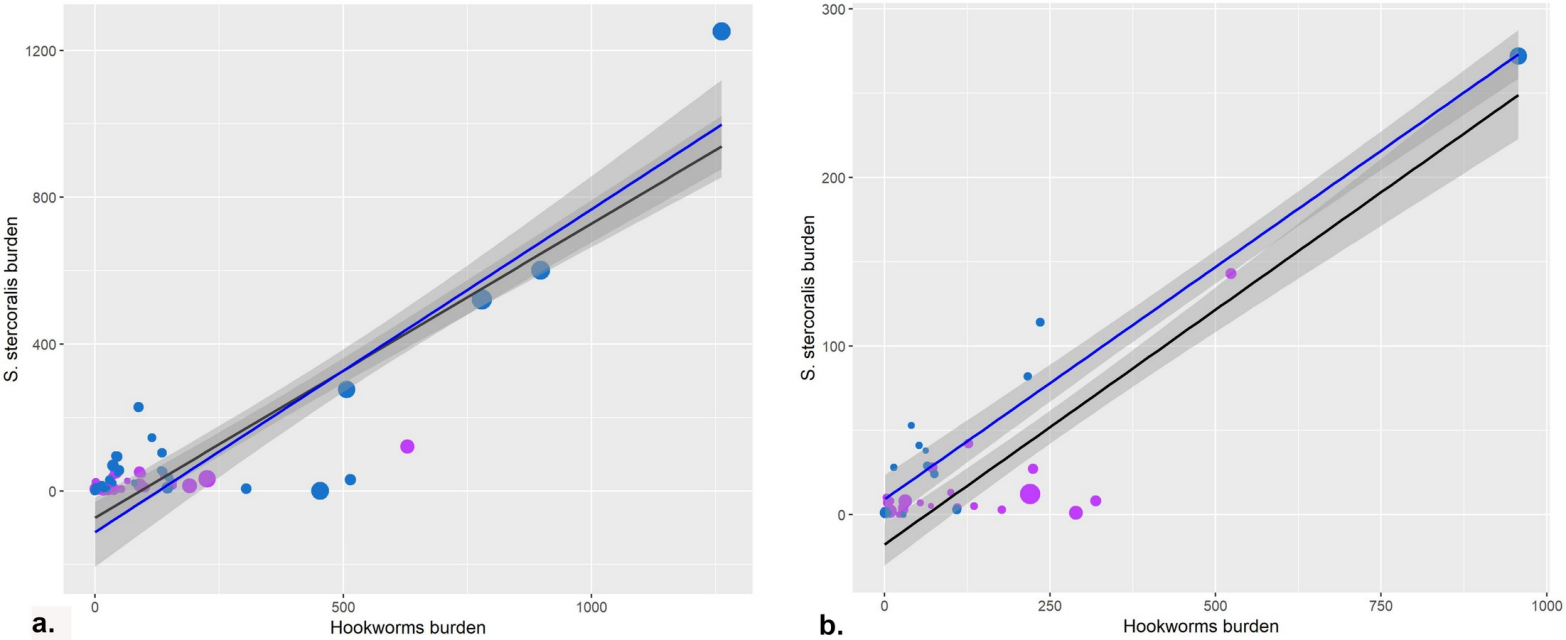
Linear regression. Blue dots represent surveys with high sensitivity diagnostic methods (Hight-S) and purple dots represent surveys with low sensitivity diagnostic methods (Low-S). **a. Community surveys.** The black line represents the curve of equation 1 (Table 1), for the linear regression of all surveys (Hight-S and Low-S) of the Community population. The blue line represents the curve of equation 2 (Table 1), for the linear regression of all surveys of the Community population with Hight-S. **b. School-aged children (SAC) surveys.** The black line represents the curve of equation 3 (Table 1), for the linear regression of all surveys (Hight-S and Low-S) of the SAC population. The blue line represents the curve of equation 4 (Table 1), for the linear regression of all surveys of the SAC population with Hight-S. Gray shading represents the 95% confidence interval.

Regression models were performed for both population groups with and without a quadratic term for hookworm prevalence and adjusting for diagnostic sensitivity (Hight-S and Low-S) and WHO region (African Region, Eastern Mediterranean Region, European Region, Region of the Americas, South-East Asian Region and Western Pacific Region). For both populations, the model with a quadratic term for the prevalence of hookworm did not have a better fit than the linear model and it was superior using High-S surveys than all surveys. In addition, cross-validation showed better results for both populations (Community and SAC) using High-S surveys. Thus, we were able to estimate the burden of *S*. *stercoralis* for Community and SAC population according to the following equations (Fig 5):

*St*_*com_burden*_ = = -112.42585 + 0.88005*HKW_*com_burden*_ (2) (Table 1)

And

*St*_*SAC_burden*_ = 9.0354 + 0.2759*HKW_*SAC_burden*_ (4) (Table 1)

**Table 1:** Linear equations for the different populations. The total and school-aged children (SAC) population infected with hookworms and the proportion of SAC in each region were obtained from References 12 and 21. St: *Strongyloides stercoralis*, HKW: hookworm, Com_burden: Community burden, SAC_burden: SAC burden, High-S: high sensitivity methods, 95%CI: 95% confidence interval.

Population	Equation (Equation number)	P-value of the slope coefficient	R^2^	Infected population with hookworms (millions)	Estimated infected population (millions) with *S*. *stercoralis* (95%CI)
Community	*St*_*com_burden*_ = -73.26879 + 0.80169*HKW_*com_burden*_ (1)	2*10^−16^	0.82		
Community + High-S	*St*_*com_burden*_ = -112.42585 + 0.88005*HKW_*com_burden*_ (2)	3*10^−14^	0.83	**World:** 438.9	386 (324–449)
				**Asia:**281.8	248 (206–288)
				**Africa:**117.7	104 (87–120)
				**America:**30.3	27 (22–31)
SAC	*St*_*SAC_burden*_ = -17.7371 + 0.2786*HKW_*SAC_burden*_ (3)	2*10^−16^	0.87
SAC + High-S	*St*_*SAC_burden*_ = 9.0354 + 0.2759*HKW_*SAC_burden*_ (4)	4*10^−12^	0.98	**World:** 438.9*0.1815 = 79.7	22 (20–24)
				**Asia:** 281.8*0.1573 = 44.3	12 (11–13)
				**Africa:** 117.7*0.2623 = 30.9	8 (7–9)
				**America:**30.3*0.1725 = 5.2	14 (13–15)

### Estimated prevalence of *S*. *stercoralis*

Based on the estimated regression coefficients and the estimate of population infected by hookworms of 438.9 million [12], we were able to estimate the prevalence of *S*. *stercoralis* in 386 million (95%CI 324–449 million) people worldwide. In turn, based on these global numbers and the proportion of SAC worldwide [21], we were able to estimate 22 million (95%CI 20–24 million) SAC infected with *S*. *stercoralis* globally.

## Discussion

This systematic review of the literature adds further evidence through an innovative approach to the relationship between hookworms and *S*. *stercoralis*, which share biologic and epidemiologic features as the host age distribution, infection route, and risk factors [9,22]. Results suggest that the burden of infections by hookworms can be used as a proxy to estimate the burden of individuals infected by *S*. *stercoralis*, which is consistent with previous hypothesis [11]. This positive correlation is obtained from a dataset that includes a wide variety of surveys from different geographic regions (Fig 3) performed with different diagnostic methods and is stronger when using a subset of surveys that included in their diagnostic approach laboratory techniques of higher sensitivity for *S*. *stercoralis*. While this is encouraging, most of these techniques, including those using molecular biology methods, are still sub-optimal, likely resulting in an underestimated real prevalence of *S*. *stercoralis* [23,24].

The diagnosis of hookworm infection, although simpler and with higher sensitivity than for *S*. *stercoralis*, is also subject to underestimations due to limitations of the most frequently used techniques, which have an estimated sensitivity of 71 to 78% for high intensity hookworm infections in the 2-slide Kato Katz method and drops to 46 to 60% in low intensity infections [25], a sensitivity that might be even lower when delays occur between sample acquisition and reading [26]. Despite these relevant diagnostic limitations that impact these two species differently, the positive correlation remains significant.

The mechanisms underlying the relationship have not been explored in this analysis and are possibly related to a shared infection route: mainly the lack of adequate sanitation as a common risk factor for both species. This contrasts with *A*. *lumbricoides*, and *T*. *trichiura*, which have a different and also shared infection route and have been linked to lack of adequate water supply [9]. Interestingly, *A*. *lumbricoides*, and *T*. *trichiura* have been identified by other authors as having a significant correlation and closely related distribution across surveys in different locations with high and low prevalence, that was not observed for hookworms, which showed a distribution independent of the other species [27]. These observations are consistent and in line with our findings. Specifically, while we found a positive strong correlation between hookworms, and *S*. *stercoralis* burdens, a weaker correlation was found between *S*. *stercoralis* and *A*. *lumbricoides* or *T*. *trichiura*. In addition, consistent with previous reports, our study also shows a strong correlation between the two latter species [27].

The relationship between *S*. *stercoralis* and hookworms acquires relevance as a consequence of the renewed interest in incorporating the former into the WHO leaded strategy for the control and elimination of STH [4]. While the role of strongyloidiasis in public health has had a growing interest in the recent years [11,28], only its incorporation into an ongoing and successful implementation program, as is WHO`s strategy, puts strongyloidiasis as an achievable goal for control. This recognition as a candidate for incorporation into the STH control strategy, opens two key questions on *S*. *stercoralis*; (1) what is the size of the global burden? and (2) what adjustments are needed in the current anthelmintic regimens?

Estimates of the burden of *S*. *stercoralis* infections have been the subject of different efforts, all limited by the difficulties in diagnostics and worsened by reports of surveys using inadequate diagnostic methods [28]. Those reports inform a wide range of estimated infected individuals ranging from 3 to 100 million [7,29]. For the purpose of intervention activities, in view of the current target groups for preventive chemotherapy through MDA campaigns, the number of SAC at-risk appears as a necessary piece of information. Based on the approach taken in this analysis, these numbers are conservatively estimated at 386 million individuals worldwide infected with *S*. *stercoralis*, including 22 million school-aged children.

While the search of correlations between the different species of STH stimulated the search for combined control with drugs active against all of them as is considered to be the case for albendazole and mebendazole [27,30], these drugs appear as unlikely to achieve elimination goals due to their poor efficacy mainly against *T*. *trichiura* [31]. Drug combinations including ivermectin are a prime candidate to improve efficacy against *T*. *trichiura* while also including *S*. *stercoralis* in the spectrum of activity of the pharmacologic regimens against STH, therefore making elimination goals feasible based on spectrum and efficacy.

A number of limitations should be considered when interpreting results from this study. First, our approach did not explore co-infections between hookworms and *S*. *stercoralis* as has been shown by other authors but rather the co-endemicity status between these STH [22]. Also, the estimates of this study are limited to baseline surveys, before chemotherapeutic interventions, which can disrupt the relationship between species as they do with different epidemiologic aspects of these infections [32]. The diagnostic accuracy of the methods used across surveys is probably the biggest limitation, affecting the reported prevalence rates of *S*. *stercoralis* more than those of hookworms; an effect that is likely to be responsible for the stronger correlations in the subset of surveys that used diagnostic methods of relatively higher sensitivity. Another limitation is that the equations represent linear functions which might not fully explain the complex relationship between these 2 species. Still, other regressions failed to better explain the variability of *S*. *stercoralis* burden. Also, the global and regional burden of hookworm infections was obtained from another estimation which carries its own limitation [12], although acknowledged by the most recent guidelines by WHO [33]. Finally, the potentially different correlation between *S*. *stercoralis* with *A*. *duodenale* and *N*. *americanus* has not been explored since the large majority of surveys reported hookworms as a group, preventing the analysis by separate species. Taking these limitations into account, we were able to find two equations, one for whole communities and one for SAC, for the estimation of the burden of *S*. *stercoralis* using the burden of hookworms as an independent variable. According to this results, confirmed by a cross validation, we can state that although it is not possible to fully explain the *S*. *stercoralis* burden just with hookworm burden, the strong correlations obtained for both types of surveys offers an evidence based approximation to understand the size of the population infected by *S*. *stercoralis*; and that the use of diagnostic approaches of higher sensitivity, allows a reasonable estimate of the prevalence of *S*. *stercoralis* in the absence of a better model. It should even be noted that the estimated values of *S. stercoralis* could have been underestimated if it is taken into account that the burden of hookworm may also be underestimated due to the use of low sensitivity diagnostic techniques.

In conclusion, the significant and consistent relationship between *S*. *stercoralis* and hookworms found in our analysis suggests that a simple equation based on the hookworm prevalence could be used as an approximation to estimate the global burden of *S*. *stercoralis* infections in most although not all epidemiologic settings. The inclusion of *S*. *stercoralis* in STH control programs can be strenghthened by an approach targeting both parasites since they share the route of infection, risk factors and age distribution.

## Supporting information

S1 Table PRISMA checklist. (PDF)

S2 Table GATHER checklist. (PDF)

S3 Table Articles included in the systematic review. (XLSX)

S1 File Example of the search strategy. (PDF)

S2 File References of the articles included in the systematic review. (PDF)
