# Supplementary material for: The Strongyloides stercoralis-hookworms association as a path to the estimation of the global burden of strongyloidiasis: A systematic review
Source: PLoS Negl Trop Dis. 2020 Apr 13;14(4):e0008184. doi: 10.1371/journal.pntd.0008184 (PMC7188296; doi:10.1371/journal.pntd.0008184)
Supplement: S2 Table — (PDF) [file pntd.0008184.s002.pdf]

S2 Table. Gather checklist

Item #	Checklist item	Reported on page #
Objectives and funding		
**1**	Define the indicator(s), populations (including age, sex, and geographic entities), and time period(s) for which estimates were made.	Methods: paragraphs 2 to 6
**2**	List the funding sources for the work.	N/A
Data Inputs		
For all data inputs from multiple sources that are synthesized as part of the study:		
**3**	Describe how the data were identified and how the data were accessed.	Methods: paragraph 1
**4**	Specify the inclusion and exclusion criteria. Identify all ad-hoc exclusions.	MethodS: paragraph 2 to 5
**5**	Provide information on all included data sources and their main characteristics. For each data source used, report reference information or contact name/institution, population represented, data collection method, year(s) of data collection, sex and age range, diagnostic criteria or measurement method, and sample size, as relevant.	Supporting Information : S4 and S5

**6**	Identify and describe any categories of input data that have potentially important biases (e.g., based on characteristics listed in item 5).	Methods: paragraph 8
For data inputs that contribute to the analysis but were not synthesized as part of the study:		
**7**	Describe and give sources for any other data inputs.	N/A
For all data inputs:		
8	1. Provide all data inputs in a file format from which data can be efficiently extracted (e.g., a spreadsheet rather than a PDF), including all relevant meta-data listed in item 5. For any data inputs that cannot be shared because of ethical or legal reasons, such as third-party ownership, provide a contact name or the name of the institution that retains the right to the data.	Supporting Information : S4
Data analysis		
**9**	Provide a conceptual overview of the data analysis method. A diagram may be helpful.	Method, paragraphs 9 and 10
**10**	Provide a detailed description of all steps of the analysis, including mathematical formulae. This description should cover, as relevant, data cleaning, data pre-processing, data adjustments and weighting of data sources, and mathematical or statistical model(s).	Method, paragraphs 9 and 10
**11**	Describe how candidate models were evaluated and how the final model(s) were selected.	N/A
**12**	Provide the results of an evaluation of model performance, if done, as well as the results of any relevant sensitivity analysis.	N/A

**13**	Describe methods for calculating uncertainty of the estimates. State which sources of uncertainty were, and were not, accounted for in the uncertainty analysis.	N/A
**14**	State how analytic or statistical source code used to generate estimates can be accessed.	N/A
Results and Discussion		
**15**	Provide published estimates in a file format from which data can be efficiently extracted.	Results Table 1
**16**	Report a quantitative measure of the uncertainty of the estimates (e.g. uncertainty intervals).	Results Table 1
**17**	Interpret results in light of existing evidence. If updating a previous set of estimates, describe the reasons for changes in estimates.	Discussion paragraphs 4 and 5
**18**	Discuss limitations of the estimates. Include a discussion of any modelling assumptions or data limitations that affect interpretation of the estimates.	Discussion paragraph 7
