# Supplementary material for: The Strongyloides stercoralis-hookworms association as a path to the estimation of the global burden of strongyloidiasis: A systematic review
Source: PLoS Negl Trop Dis. 2020 Apr 13;14(4):e0008184. doi: 10.1371/journal.pntd.0008184 (PMC7188296; doi:10.1371/journal.pntd.0008184)
Supplement: S1 File — (PDF) [file pntd.0008184.s004.pdf]

S1 File. Example of the search strategy

Search date: 6 June 2018

Database: Ovid MEDLINE(R) Epub Ahead of Print, In-Process & Other Non-Indexed Citations,
Ovid MEDLINE(R) Daily and Ovid MEDLINE(R) <1946 to Present>

Search Strategy:

----

1 (strongyloid* or S stercoralis or estrongyloid* or estrongiloid*).mp. (6269)
2 (hook worm or hook-worm or hookworm or ancylostom* or necator* or uncinaria).mp. (8490)
3 exp STRONGYLOIDES/ or exp STRONGYLOIDES STERCORALIS/ (2302)
4 exp ANCYLOSTOMATOIDEA/ (2385)
5 1 or 3 (6269)
6 2 or 4 (8490)
7 5 and 6 (832)

8 limit 7 to yr="1988 -Current" (506)
9 7 and 8 (506) Search date: 6 June 2018
Database: Ovid MEDLINE(R) Epub Ahead of Print, In-Process & Other Non-Indexed Citations,
Ovid MEDLINE(R) Daily and Ovid MEDLINE(R) <1946 to Present
