# Supplementary material for: The Strongyloides stercoralis-hookworms association as a path to the estimation of the global burden of strongyloidiasis: A systematic review
Source: PLoS Negl Trop Dis. 2020 Apr 13;14(4):e0008184. doi: 10.1371/journal.pntd.0008184 (PMC7188296; doi:10.1371/journal.pntd.0008184)
Supplement: S2 File — (PDF) [file pntd.0008184.s005.pdf]

S2 File. References of the articles included in the systematic review.

1. Waree P, Polseela P, Pannarunothai S, Pipitgool V. The present situation of paragonimiasis in endemic area in Phitsanulok Province. Southeast Asian J Trop Med Public Health. 2001;32 Suppl 2:51–4.
2. Carme B, Motard A, Bau P, Day C, Aznar C, Moreau B. Intestinal parasitoses among Wayampi Indians from French Guiana. Parasite. 2002;9(2):167–74.
3. Randall AE, Perez MA, Floyd S, Black GF, Crampin AC, Ngwira B, et al. Patterns of helminth infection and relationship to BCG vaccination in Karonga District, northern Malawi. Trans R Soc Trop Med Hyg. 2002;96(1):29–33.
4. Cerdas C, Araya E, Coto S. Parásitos intestinales en la Escuela 15 de agosto, Tirrases de Curridabat, Costa Rica. Mayo-Junio de 2002. Rev Costarric Cienc Med. 2003;24(3/4):127–33.
5. Cooper PJ, Chico ME, Gaus D, Griffin GE. Relationship between bacille Calmette-Guerin vaccination, Mantoux test positivity, and geohelminth infection. Trans R Soc Trop Med Hyg. 2003;97(4):473–6.
6. Faulkner CT, Garcia BB, Logan MH, New JC, Patton S. Prevalence of endoparasitic infection in children and its relation with cholera prevention efforts in Mexico. Pan Am J Public Heal. 2003;14(1):31–41.
7. Fontes G, Lessa Oliveira KK, Lessa Oliveira AK, Mauricio da Rocha EM. Influence of specific treatment of intestinal parasites and schistosomiasis on prevalence in students in Barra de Santo Antonio, AL. [Portuguese]. Rev Soc Bras Med Trop. 2003;36(5):625–8.
8. Marcos L, Maco V, Terashima A, Samalvides F, Miranda E, Gotuzzo E. Parasitosis intestinal en poblaciones urbana y rural en Sandia, Departamento de Puno, Perú. Parasitol Latinoam. 2003;58(1–2):35–40.
9. Nithikathkul C, Changsap B, Wannapinyosheep S, Arnat N, Kongkham S, Benchawattananon R, et al. Parasitic infections among Karen in Kanchanaburi Province, western Thailand. Southeast Asian J Trop Med Public Heal. 2003;34 Suppl 2:86–9.
10. Oliveira MC, Da Silva C V, Costa-Cruz JM. Intestinal parasites and commensals among individuals from a landless camping in the rural area of Uberlandia, Minas Gerais, Brazil. Rev Inst Med Trop Sao Paulo. 2003;45(3):173–6.
11. Sithithaworn P, Srisawangwong T, Tesana S, Daenseekaew W, Sithithaworn J, Fujimaki Y, et al. Epidemiology of Strongyloides stercoralis in north-east Thailand: application of the agar plate culture technique compared with the enzyme-linked immunosorbent assay. Trans R Soc Trop Med Hyg. 2003;97(4):398–402.
12. Tang N, Luo NJ. A cross-sectional study of intestinal parasitic infections in a rural district of west China. Can J Infect Dis. 2003;14(3):159–62.
13. Taranto NJ, Cajal SP, Marzi MC de, Fernández MM, Frank FM, Brú AM, et al. Clinical status and parasitic infection in a Wichí Aboriginal community in Salta, Argentina. Trans R Soc Trop Med Hyg. 2003;97(5):554–8.
14. Aimpun P, Hshieh P. Survey for intestinal parasites in Belize, Central America. Southeast Asian J Trop Med Public Heal. 2004;35(3):506–11.
15. Anosike JC, Nwoke BE, Onwuliri CO, Obiukwu CE, Duru AF, Nwachukwu MI, et al. Prevalence of parasitic diseases among nomadic Fulanis of south-eastern Nigeria. Ann Agric Environ Med. 2004;11(2):221–5.
16. Ibáñez H N, Jara C C, Guerra M A, Díaz L E. Prevalencia del Enteroparasitismo en escolares de comunidades nativas del Alto Marañon, Amazonas, Perú. Rev Peru Med Exp Salud Publica. 2004;21(3):126–33.

17. Koga-Kita K. Intestinal parasitic infections and socioeconomic status in Prek Russey Commune, Cambodia. Nippon Koshu Eisei Zasshi - Japanese J Public Heal. 2004;51(11):986–92.
18. Mengistu L, Berhanu E. Prevalence of intestinal parasites among schoolchildren in a rural area close to the southeast of Lake Langano, Ethiopia. Ethiop J Heal Dev. 2004;18(2):116–20.
19. Saksirisampant W, Prownebon J, Kanmarnee P, Thaisom S, Yenthakam S, Nuchprayoon S. Prevalence of parasitism among students of the Karen hill-tribe in Mae Chame District, Chiang Mai Province, Thailand. J Med Assoc Thail. 2004;87(SUPPL. 2):S278–83.
20. Singh HL, Singh NB, Singh YI. Helminthic infestation of the primary school-going children in Manipur. J Commun Dis. 2004;36(2):111–6.
21. Araujo CF, Fernández CL. Prevalência de parasitoses intestinais na cidade de Eirunepé, Amazonas. Rev Soc Bras Med Trop. 2005;38(1):69.
22. Champetier de Ribes G, Fline M, Désormeaux AM, Eyma E, Montagut P, Champagne C, et al. Helminthoses intestinales en milieu scolaire en Haïti en 2002. Bull Soc Pathol Exot. 2005;98(2):127–32.
23. Chessed G, Kwalagbe B, Furo NA. Intestinal helminthiases among school children in Gyawana District, Adamawa State, Nigeria. Glob J Pure Appl Sci. 2005;11(2):193–6.
24. Girum T. The prevalence of intestinal helminthic infections and associated risk factors among school children in Babile town, eastern Ethiopia. Ethiop J Heal Dev. 2005;19(2):140–7.
25. Hernandez ChavarrÍA F, Matamoros Madrigal MF. Parásitos intestinales en una comunidad Amerindia, Costa Rica: COSTA RICA. Parasitol Latinoam. 2005;60(3–4):182–5.
26. Yelifari L, Bloch P, Magnussen P, van Lieshout L, Dery G, Anemana S, et al. Distribution of human Oesophagostomum bifurcum, hookworm and Strongyloides stercoralis infections in northern Ghana. Trans R Soc Trop Med Hyg. 2005;99(1):32–8.
27. Anosike JC, Zaccheaus VO, Adeiyongo CM, Abanobi OC, Dada EO, Oku EE, et al. Studies on the intestinal worm (helminthiasis) infestation in a Central Nigerian rural community. J Appl Sci Environ Manag. 2006;10(2):61–6.
28. Corrales LF, Izurieta R, Moe CL. Association between intestinal parasitic infections and type of sanitation system in rural El Salvador. Trop Med Int Heal. 2006;11(12):1821–31.
29. Ijagbone IF, Olagunju TF. Intestinal helminth parasites in school children in Iragbiji, Boripe Local Government, Osun State, Nigeria. African J Biomed Res. 2006;9(1):63–5.
30. Khampitak T, Knowles J, Yongvanit P, Sithithaworn P, Tangrassameeprasert R, Boonsiri P, et al. Thiamine deficiency and parasitic infection in rural Thai children. Southeast Asian J Trop Med Public Health. 2006;37(3):441–5.
31. Patel PK, Khandekar R. Intestinal parasitic infections among school children of the Dhahira Region of Oman. Saudi Med J. 2006;27(5):627–32.
32. Sithithaworn P, Sukavat K, Vannachone B, Sophonphong K, Ben-Embarek P, Petney T, et al. Epidemiology of food-borne trematodes and other parasite infections in a fishing community on the Nam Ngum reservoir, Lao PDR. Southeast Asian J Trop Med Public Heal. 2006;37(6):1083–90.
33. Yori PP, Kosek M, Gilman RH, Cordova J, Bern C, Chavez CB, et al. Seroepidemiology of strongyloidiasis in the Peruvian Amazon. Am J Trop Med Hyg. 2006;74(1):97–102.
34. Aguiar JIA, Goncalves AQ, Sodre FC, Pereira SDR, Boia MN, De Lemos ERS, et al. Intestinal protozoa and helminths among Terena Indians in the State of Mato Grosso do Sul: High prevalence of Blastocystis hominis. Rev Soc Bras Med Trop. 2007;40(6):631–4.

35. Jombo GT, Egah DZ, Akosu JT, Mbaawuaga EW. Human intestinal parasitism in a rural settlement of northern Nigeria, a survey. African J Clin Exp Microbiol. 2007;8(1):48–54.
36. Menghi CI, Iuvaro FR, Dellacasa MA, Gatta CL. Survey of intestinal parasites among an aboriginal community in Salta. Med (Buenos Aires). 2007;67(6/2):705–8.
37. Ugbomoiko US, Ofoezie IE. Multiple infection diagnosis of intestinal helminthiasis in the assessment of health and environmental effect of development projects in Nigeria. J Helminthol. 2007;81(3):227–31.
38. Warunee N, Choomanee L, Sataporn P, Rapeeporn Y, Nuttapong W, Sompong S, et al. Intestinal parasitic infections among school children in Thailand. Trop Biomed. 2007;24(2):83–8.
39. Erlanger TE, Sayasone S, Krieger GR, Kaul S, Sananikhom P, Tanner M, et al. Baseline health situation of communities affected by the Nam Theun 2 hydroelectric project in central Lao PDR and indicators for monitoring. Int J Environ Health Res. 2008;18(3):223–42.
40. Ibidapo CA, Okwa O. The prevalence and intensity of soil transmitted helminths in a rural community, Lagos suburb, South West Nigeria. Int J Agric Biol. 2008;10(1):89–92.
41. Kitvatanachai S, Boonslip S, Watanasatitarpa S. Intestinal parasitic infections in Srimum suburban area of Nakhon Ratchasima Province, Thailand. Trop Biomed. 2008;25(3):237–42.
42. Machado ER, Santos DS, Costa-Cruz JM. Enteroparasites and commensals among children in four peripheral districts of Uberlandia, State of Minas Gerais. Rev Soc Bras Med Trop. 2008;41(6):581–5.
43. Chukwuma MC, Ekejindu IM, Agbakoba NR, Ezeagwuna DA, Anaghalu IC, Nwosu DC. The prevalence and risk factors of geohelminth infections among primary school children in Ebenebe Town, Anambra State, Nigeria. Middle East J Sci Res. 2009;4(3):211–5.
44. Gamboa MI, Kozubsky LE, Costas ME, Garraza M, Cardozo MI, Susevich ML, et al. Asociación entre geohelmintos y condiciones socioambientales en diferentes poblaciones humanas de Argentina. Rev Panam Salud Publica. 2009;26(1):1–8.
45. Hassan AO, Amoo A, Hassan RO. Helminthiasis among school age children in Osogbo municipality, Osun State, Nigeria. Niger J Heal Biomed Sci. 2009;8(1):unpaginated.
46. Palhano-Silva CS, Araújo AJG, Lourenço AEP, Bastos OMP, Santos R V, Coimbra Júnior CEA. Intestinal parasitic infection in the Suruí Indians, Brazilian Amazon. Interciencia. 2009;34(4):259–64.
47. Sayasone S, Vonghajack Y, Vanmany M, Rasphone O, Tesana S, Utzinger J, et al. Diversity of human intestinal helminthiasis in Lao PDR. Trans R Soc Trop Med Hyg. 2009;103(3):247–54.
48. Tanner S, Leonard WR, McDade TW, Reyes-Garcia V, Godoy R, Huanca T. Influence of helminth infections on childhood nutritional status in lowland Bolivia. Am J Hum Biol. 2009;21(5):651–6.
49. Tilahun T. Intestinal parasitosis among Kara and Kwego semi-pastoralist tribes in lower Omo Valley, Southwestern Ethiopia. Ethiop J Heal Dev. 2009;23(1):57–62.
50. Feleke M, Yeshambel B, Moges T, Yenew K, Andargachew M, Afework K, et al. Comparison of formol-acetone concentration method with that of the direct iodine preparation and formol-ether concentration methods for examination of stool parasites. Ethiop J Heal Dev. 2010;24(2):148–51.
51. Glinz D, Silue KD, Knopp S, Lohourignon LK, Yao KP, Steinmann P, et al. Comparing diagnostic accuracy of Kato-Katz, Koga agar plate, ether-concentration, and FLOTAC for Schistosoma mansoni and soil-transmitted helminths. PLoS Neglected Trop Dis [electronic Resour. 2010;4(7):e754.

52. Lemlem L, Berhanu E, Asrat H. Current status of intestinal schistosomiasis and soil-transmitted helminthiasis among primary school children in Adwa Town, northern Ethiopia. Ethiop J Heal Dev. 2010;24(3):191–7.
53. Machado ER, Freitas CV de, Costa-Cruz JM. Strongyloides stercoralis and other enteroparasites in individuals of rural area of Uberlândia, Minas Gerais State, Brazil. Rev Patol Trop. 2010;39(2):115–22.
54. Becker L, Sieto B, Silue KD, Adjossan L, Kone S. Diagnosis , Clinical Features , and Self-Reported Morbidity of Strongyloides stercoralis and Hookworm Infection in a Co-Endemic Setting. 2011;5(8).
55. Devi U, Borkakoty B, Mahanta J. Strongyloidiasis in Assam, India: A community-based study. Trop Parasitol. 2011;1(1):30–2.
56. Guidetti C, Ricci L, Vecchia L. [Aetiology of intestinal parasites in a sample of students from Mozambique]. Infez Med. 2011;19(3):157–65.
57. Popruk S, Thima K, Udonsom R, Rattaprasert P, Sukthana Y. Does silent giardia infection need any attention? Open Trop Med J. 2011;4(1):26–32.
58. Sayasone S, Mak TK, Vanmany M, Rasphone O, Vounatsou P, Utzinger J, et al. Helminth and intestinal protozoa infections, multiparasitism and risk factors in Champasack Province, Lao People's Democratic Republic. PLoS Negl Trop Dis. 2011;5 (4) (no(e1037).
59. Traore SG, Odermatt P, Bonfoh B, Utzinger J, Aka ND, Adoubryn KD, et al. No Paragonimus in high-risk groups in Cote d'Ivoire, but considerable prevalence of helminths and intestinal protozoon infections. Parasites and Vectors. 2011;4 (1) (no(96).
60. Uhuo AC, Odikamnoro OO, Ani OC. The incidence of intestinal nematodes in primary school children in Ezza North Local Government Area, Ebonyi State Nigeria. Adv Appl Sci Res. 2011;2(5):257–62.
61. Adikankwu OR, Odikamnoro OO, Uhuo AC, Nwuzo AC. The prevalence of intestinal nematode in school children in Ebonyi local government area, Ebonyi State, Nigeria. Cont J Biomed Sci. 2012;6(1):13–7.
62. Brandelli CLC, Carli GA de, Macedo AJ, Tasca T. Intestinal parasitism and socio-environmental factors among Mbyá-Guarani indians, Porto Alegre, Rio Grande do Sul, Brazil. Rev Inst Med Trop Sao Paulo. 2012;54(3):119–22.
63. Carvalho GLX de, Moreira LE, Pena JL, Marinho CC, Bahia MT, Machado-Coelho GLL. A comparative study of the TF-Test®, Kato-Katz, Hoffman-Pons-Janer, Willis and Baermann-Moraes coprologic methods for the detection of human parasitosis. Mem Inst Oswaldo Cruz. 2012;107(1):80–4.
64. Choubisa SL, Jaroli VJ, Choubisa P, Mogra N. Intestinal parasitic infection in Bhil tribe of Rajasthan, India. J Parasit Dis. 2012;36(2):143–8.
65. Conlan J V, Khamlome B, Vongxay K, Elliot A, Pallant L, Sripa B, et al. Soil-transmitted helminthiasis in Laos: a community-wide cross-sectional study of humans and dogs in a mass drug administration environment. Am J Trop Med Hyg. 2012;86(4):624–34.
66. Ludwig KM, Ribeiro ALT, Conte A de OC, Decleva DV, Ribeiro JTD. Ocorrência de enteroparasitoses na população de um bairro da cidade de Cândido Mota-SP. J Heal Sci Inst. 2012;30(3).
67. Machicado JD, Marcos LA, Tello R, Canales M, Terashima A, Gotuzzo E. Diagnosis of soil-transmitted helminthiasis in an Amazonic community of Peru using multiple diagnostic techniques. Trans R Soc Trop Med Hyg. 2012;106(6):333–9.
68. Abate A, Kibret B, Bekalu E, Abera S, Teklu T, Yalew A, et al. Cross-Sectional Study on the Prevalence of Intestinal Parasites and Associated Risk Factors in Teda Health Centre, Northwest Ethiopia. ISRN Parasitol. 2013;2013:757451.

69. Assis EM de, Olivieria RC de, Moreira LE, Pena JL, Rodrigues LC, Machado-Coelho GLL. Prevalence of intestinal parasites in the Maxakali indigenous community in Minas Gerais, Brazil, 2009. Cad Saude Publica. 2013;29(4):681–90.
70. Babatunde SK, Adedayo MR, Ajiboye AE, Sunday O, Ameen N. Soil-transmitted helminth infections among school children in rural communities of Moro Local Government Area, Kwara State, Nigeria. African J Microbiol Res. 2013;7(45):5148–53.
71. Bayeh A, Genetu A, Mulat Y, Herrador Z. Epidemiology of soil-transmitted helminths, schistosoma mansoni, and haematocrit values among schoolchildren in Ethiopia. J Infect Dev Ctries. 2013;7(3):253–60.
72. Boonjaraspinyo S, Boonmars T, Kaewsamut B, Ekobol N, Laummaunwai P, Aukkanimart R, et al. A cross-sectional study on intestinal parasitic infections in rural communities, northeast Thailand. Korean J Parasitol. 2013;51(6):727–34.
73. Briones-Chavez C, Torres-Zevallos H, Canales M, Stamato CM, O'Riordan TG, Terashima A. Differences in prevalence of geohelminth infections between indigenous and settler populations in a remote Amazonian region of Peru. Trop Med Int Heal. 2013;18(5):615–8.
74. Damazio SM, Lima MS, Soares AR, de Souza MAA. Intestinal parasites in a quilombola community of the Northern State of Espirito Santo, Brazil. Rev Inst Med Trop Sao Paulo. 2013;55(3):179–83.
75. Hartini Y, Geishamimi G, Mariam AZ, Mohamed-Kamel AG, Hidayatul FO, Ismarul YI. Distribution of intestinal parasitic infections amongst aborigine children at Post Sungai Rual, Kelantan, Malaysia. Trop Biomed. 2013;30(4):596–601.
76. Khieu V, Schar F, Marti H, Sayasone S, Duong S, Muth S, et al. Diagnosis, treatment and risk factors of Strongyloides stercoralis in schoolchildren in Cambodia. PLoS Neglected Trop Dis [electronic Resour. 2013;7(2):e2035.
77. Schar F, Odermatt P, Khieu V, Panning M, Duong S, Muth S, et al. Evaluation of real-time PCR for Strongyloides stercoralis and hookworm as diagnostic tool in asymptomatic schoolchildren in Cambodia. Acta Trop. 2013;126(2):89–92.
78. Wegayehu T, Tsalla T, Seifu B, Teklu T. Prevalence of intestinal parasitic infections among highland and lowland dwellers in Gamo area, South Ethiopia. BMC Public Health. 2013;13:151.
79. Bracho M A, Rivero-Rodríguez Z, Rios P M, Atencio T R, Villalobos P R, Rodríguez L. Parasitosis intestinales en niños y adolescentes de la etnia Yukpa de Toromo, estado Zulia, Venezuela: Comparación de los años 2002 Y 2012. Kasmera. 2014;42(1):41–51.
80. Couto LD, Tibirica SHC, Pinheiro IO, Mitterofhe A, Lima AC, Castro MF, et al. Neglected tropical diseases: Prevalence and risk factors for schistosomiasis and soil-transmitted helminthiasis in a region of Minas Gerais State, Brazil. Trans R Soc Trop Med Hyg. 2014;108(6):363–71.
81. Khieu V, Schar F, Marti H, Bless PJ, Char MC, Muth S, et al. Prevalence and risk factors of Strongyloides stercoralis in Takeo Province, Cambodia. Parasites Vectors [Electronic Resour. 2014;7:221.
82. Neres-Norberg A, Guerra-Sanches F, Blanco Moreira-Norberg PR, Madeira-Oliveira JT, Santa-Helena AA, Serra-Freire NM. Enteroparasitismo en Indígenas Terena en el Estado de Mato Grosso do Sul, Brasil. Rev Salud Publica. 2014;16(6):859–70.
83. Pino Santos A, Nunez Fernandez FA, Martinez Sanchez R, Domenech Canete I, Rodriguez M, Jerez Puebla L, et al. Prevalence and risk factors for intestinal parasitic infections in a rural community in "Consolacion del Sur" municipality, Cuba. West Indian Med J. 2014;63(4):333–9.
84. Schar F, Inpankaew T, Traub RJ, Khieu V, Dalsgaard A, Chimnoi W, et al. The prevalence and diversity of intestinal parasitic infections in humans and domestic animals in a rural Cambodian village. Parasitol Int. 2014;63(4):597–603.

85. Abah AE, Arene FO. Status of Intestinal Parasitic Infections among Primary School Children in Rivers State, Nigeria. J Parasitol Res. 2015;2015:937096.
86. Becker SL, Piraisoody N, Kramme S, Marti H, Silué KD, Panning M, et al. Real-time PCR for detection of Strongyloides stercoralis in human stool samples from Côte d'Ivoire: Diagnostic accuracy, inter-laboratory comparison and patterns of hookworm co-infection. Acta Trop. 2015;150:210–7.
87. Cardozo Ocampos GE, Cañete Duarte Z, Lenartovicz V. Frecuencia de enteroparásitos en niños y niñas del primer ciclo de la educación escolar básica de escuelas públicas de ciudad del este, Paraguay. Mem Inst Invest Cienc Salud. 2015;13(1):24–30.
88. Cimino RO, Jeun R, Juarez M, Cajal PS, Vargas P, Echazu A, et al. Identification of human intestinal parasites affecting an asymptomatic peri-urban Argentinian population using multi-parallel quantitative real-time polymerase chain reaction. Parasites Vectors [Electronic Resour. 2015;8:380.
89. Frickmann H, Schwarz NG, Rakotozandrindrainy R, May J, Hagen RM. PCR for enteric pathogens in high-prevalence settings. What does a positive signal tell us? Infect Dis (Auckl). 2015;47(7):491–8.
90. Kaewpitoon SJ, Loyd RA, Kaewpitoon N. A Cross-Sectional Survey of Intestinal Helminthiases in Rural Communities of Nakhon Ratchasima Province, Thailand. J Med Assoc Thail. 2015;98 Suppl 4:S27-32.
91. Ogochukwu CO, Patience OU. A cross-sectional study of Ascaris lumbricoides infection in a rural community in Ebonyi state, Nigeria: prevalence and risk factors. Iran J Public Health. 2015;44(10):1430–2.
92. Polseela R, Vitta A. Prevalence of intestinal parasitic infections among schoolchildren in Phitsanulok Province, Northern Thailand. Asian Pacific J Trop Dis. 2015;5(7):539–42.
93. Sandoval NR, Rios N, Mena A, Fernandez R, Perea M, Manzano-Roman R, et al. A survey of intestinal parasites including associated risk factors in humans in Panama. Acta Trop. 2015;147:54–63.
94. Shield J, Aland K, Kearns T, Gongdjalk G, Holt D, Currie B, et al. Intestinal parasites of children and adults in a remote Aboriginal community of the Northern Territory, Australia, 1994-1996. West Pacific Surveill Response J. 2015;6(1):44–51.
95. Alsubaie ASR, Azazy AA, Omer EO, Al-Shibani LA, Al-Mekhlafi AQ, Al-Khawlani FA. Pattern of parasitic infections as public health problem among school children: A comparative study between rural and urban areas. J Taibah Univ Med Sci. 2016;11(1):13–8.
96. Amor A, Rodriguez E, Saugar JM, Arroyo A, Lopez-Quintana B, Abera B, et al. High prevalence of Strongyloides stercoralis in school-aged children in a rural highland of north-western Ethiopia: the role of intensive diagnostic work-up. Parasites Vectors [Electronic Resour. 2016;9(1):617.
97. Begna T, Solomon T, Yohannes Z, Eden A. Intestinal parasitic infections and nutritional status among primary school children in Delo-Mena district, South Eastern Ethiopia. Iran J Parasitol. 2016;11(4):549–58.
98. Boko PM, Ibikounle M, Onzo-Aboki A, Tougoue JJ, Sissinto Y, Batcho W, et al. Schistosomiasis and Soil Transmitted Helminths Distribution in Benin: A Baseline Prevalence Survey in 30 Districts. PLoS ONE [Electronic Resour. 2016;11(9):e0162798.
99. Easton A V, Oliveira RG, O'Connell EM, Kepha S, Mwandawiro CS, Njenga SM, et al. Multi-parallel qPCR provides increased sensitivity and diagnostic breadth for gastrointestinal parasites of humans: field-based inferences on the impact of mass deworming. Parasites Vectors [Electronic Resour. 2016;9:38.

100. Sanprasert V, Srichaipon N, Bunkasem U, Srirungruang S, Nuchprayoon S. Prevalence of intestinal protozoan infections among children in Thailand: a large-scale screening and comparative study of three standard detection methods. Southeast Asian J Trop Med Public Health. 2016;47(6):1123–33.
101. Tork M, Sharif M, Charati JY, Nazar I, Hosseini SA. Prevalence of intestinal parasitic infections and associated risk factors in West of Mazandaran Province, Iran. [Persian]. J Maz Univ Med Sci. 2016;25(134):81–8.
102. Webb EL, Nampijja M, Kizindo R, Namutebi M, Nakazibwe E, Oduru G, et al. Helminths are positively associated with atopy and wheeze in Ugandan fishing communities: results from a cross-sectional survey. Allergy Eur J Allergy Clin Immunol. 2016;71(8):1156–69.
103. Abdi M, Nibret E, Munshea A. Prevalence of intestinal helminthic infections and malnutrition among schoolchildren of the Zegie Peninsula, northwestern Ethiopia. J Infect Public Health. 2017;10(1):84–92.
104. Dankwa K, Addy-Lamptey P, Latif A, Essien-Baidoo S, Ephraim RKD, Gavor-Kwashi CEK, et al. Intestinal parasitic infections among primary school pupils in Elmina, a fishing community in Ghana. Int J Med Heal Sci. 2017;6(3):151–7.
105. de Alegria M, Colmenares K, Espasa M, Amor A, Lopez I, Nindia A, et al. Prevalence of Strongyloides stercoralis and Other Intestinal Parasite Infections in School Children in a Rural Area of Angola: A Cross-Sectional Study. Am J Trop Med Hyg. 2017;97(4):1226–31.
106. Echazu A, Juarez M, Vargas PA, Cajal SP, Cimino RO, Heredia V, et al. Albendazole and ivermectin for the control of soil-transmitted helminths in an area with high prevalence of Strongyloides stercoralis and hookworm in northwestern Argentina: A community-based pragmatic study. PLoS Neglected Trop Dis [electronic Resour. 2017;11(10):e0006003.
107. Incani RN, Ferrer E, Hoek D, Ramak R, Roelfsema J, Mughini-Gras L, et al. Diagnosis of intestinal parasites in a rural community of Venezuela: Advantages and disadvantages of using microscopy or RT-PCR. Acta Trop. 2017;167:64–70.
108. Kitvatanachai S, Taylor A, Rhongbutsri P, Pongstaporn W. Determine the prevalence of intestinal and soil-transmitted helminths using different copromicroscopic techniques in Krabi Province, Thailand. Asian Pacific J Trop Dis. 2017;7(12):719–23.
109. McKenna ML, McAtee S, Bryan PE, Jeun R, Ward T, Kraus J, et al. Human Intestinal Parasite Burden and Poor Sanitation in Rural Alabama. Am J Trop Med Hyg. 2017;97(5):1623–8.
110. Meurs L, Polderman AM, Vinkeles Melchers N V, Brienen EA, Verweij JJ, Groosjohan B, et al. Diagnosing Polyparasitism in a High-Prevalence Setting in Beira, Mozambique: Detection of Intestinal Parasites in Fecal Samples by Microscopy and Real-Time PCR. PLoS Neglected Trop Dis [electronic Resour. 2017;11(1):e0005310.
111. Ribas A, Jollivet C, Morand S, Thongmalayvong B, Somphavong S, Siew CC, et al. Intestinal Parasitic Infections and Environmental Water Contamination in a Rural Village of Northern Lao PDR. Korean J Parasitol. 2017;55(5):523–32.
112. Senephansiri P, Laummaunwai P, Laymanivong S, Boonmar T. Status and Risk Factors of Strongyloides stercoralis Infection in Rural Communities of Xayaburi Province, Lao PDR. Korean J Parasitol. 2017;55(5):569–73.
113. Suntaravitun P, Dokmaikaw A. Prevalence of intestinal protozoan infections among schoolchildren in Bang Khla District, Chachoengsao Province, Central Thailand. Asian Pacific J Trop Dis. 2017;7(9):523–6.

114. Adu-Gyasi D, Asante KP, Frempong MT, Gyasi DK, Iddrisu LF, Ankrah L, et al. Epidemiology of soil transmitted Helminth infections in the middle-belt of Ghana, Africa. Parasite Epidemiol Control. 2018;3(3):e00071.

115. Barbosa C V, Barreto MM, Andrade RJ, Sodre F, D'Avila-Levy CM, Peralta JM, et al. Intestinal parasite infections in a rural community of Rio de Janeiro (Brazil): Prevalence and genetic diversity of Blastocystis subtypes. PLoS ONE [Electronic Resour. 2018;13(3):e0193860.

116. Forrer A, Khieu V, Schar F, Vounatsou P, Chammartin F, Marti H, et al. Strongyloides stercoralis and hookworm co-infection: spatial distribution and determinants in Preah Vihear Province, Cambodia. Parasites Vectors [Electronic Resour. 2018;11(1):33.

117. M'Bondoukwe N P, Kendjo E, Mawili-Mboumba DP, Koumba Lengongo J V, Offouga Mbouoronde C, Nkoghe D, et al. Prevalence of and risk factors for malaria, filariasis, and intestinal parasites as single infections or co-infections in different settlements of Gabon, Central Africa.[Erratum appears in Infect Dis Poverty. 2018 Apr 20;7(1):38; PMID: 29678200]. Infect Dis Poverty. 2018;7(1):6.

118. Suntaravitun P, Dokmaikaw A. Prevalence of Intestinal Parasites and Associated Risk Factors for Infection among Rural Communities of Chachoengsao Province, Thailand. Korean J Parasitol. 2018;56(1):33–9.

119. Tuyizere A, Ndayambaje A, Walker TD, Bayingana C, Ntirenganya C, Dusabejambo V, et al. Prevalence of Strongyloides stercoralis infection and other soil-transmitted helminths by cross-sectional survey in a rural community in Gisagara District, Southern Province, Rwanda. Trans R Soc Trop Med Hyg. 2018;112(3):97–102.
